# Giant colonic lipoma causing intussusception: CT scan and clinical findings

**DOI:** 10.11604/pamj.2019.32.27.18040

**Published:** 2019-01-16

**Authors:** Ozkan Ozen, Yilmaz Guler, Yavuz Yuksel

**Affiliations:** 1 Alanya Alaaddin Keykubat University, Alanya Education and Research Hospital, Department of Radiology, Antalya, Turkey; 2 Alanya Alaaddin Keykubat University Alanya Education and Research Hospital, Department of General Surgery, Antalya, Turkey

**Keywords:** Colon, computerized tomography, lipoma, liposarcoma, intussusception

## Abstract

Colonic lipomas are uncommon and usually asymptomatic tumors. A 30-year-old woman with abdominal pain lasting 10 days was admitted to the surgical clinic. Her physical examination revealed sensitivity on the right upper quadrant and her bowel sounds were normal. A lesion and invagination findings in the colon were found in the ultrasound examination and CT was performed. CT scan revealed a lipoma and invagination in the colon and the patient has undergone surgery. Pathological diagnosis of the lesion was reported as submucosallipoma. In this case report, we present clinical and radiological findings of a submucosal colonic lipoma causing intussusception.

## Introduction

Lipomas are benign nonepithelial adipose tumors that can be seen throughout the gastrointestinal tract, although they occur most frequently in the colon [1, 2]. Sice most cases are asymptomatic, diagnosis is usually made incidentally during colonoscopy, surgery or autopsy [3, 4]. Lipomas larger than 4 cm are called giant lipomas and most of them are symptomatic [5]. Uncomplicated gastrointestinal lipomas can be easily diagnosed by computerized tomography (CT) scan and Magnetic resonance imaging (MRI) [6]. In this article, we present CT scan and clinical findings of a giant submucosallipoma in the ascending colon that caused intussusception in a 30-year-old female patient.

## Patient and observation

**Figure 1:**
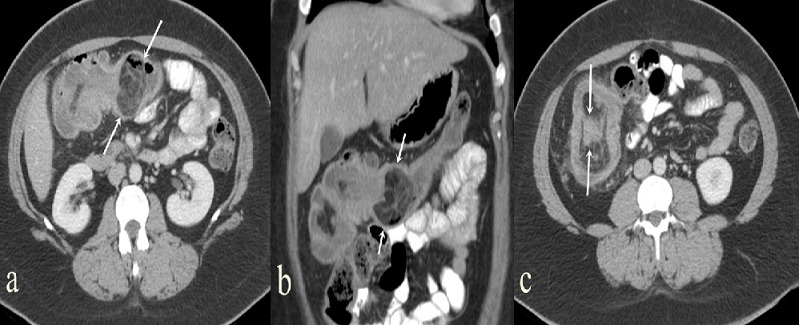
(A) in IV-Oral contrast abdominal computed tomography with axial section and coronal reformat image; (B) a mass lesion (arrows) is seen in the upper quadrant of the iliocecal junction with heterogeneous fat density and septal structures at this level. Axial view of IV-Oral contrast abdominal CT; (C) the herniation of the ileum into colonic pouch is seen (arrows)

**Figure 2:**
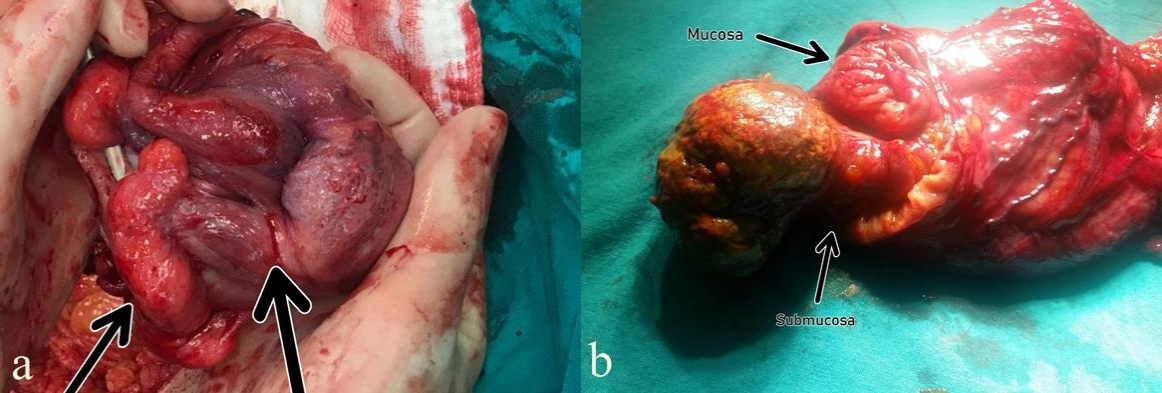
(A) during the operation, it was observed that a mass measuring approximately 6x5 cm in diameter and located about 5 cm distal to the caecum caused invagination of colon and adhesions were formed in the distal ileum; (B) when the intramuscular lumen was opened after right hemicolectomy, a mass with smooth-margins measuring about 4x5 cm and originating from submucosa was detected

A 30-year-old female patient was admitted to General Surgery outpatient clinic with complaints of abdominal pain which lasted for about 10 days and worsened during last 2 days. Abdominal pain was on both upper quadrants, especially on the right upper quadrant. Apart from abdominal pain, there was not any significant complaint (nausea, vomiting, absence of bowel movements, dysuria, etc). The patient's physical examination revealed normal blood pressure, pulse and body temperature and no abdominal distension. Tenderness was present on right upper quadrant, however, there was no rebound and bowel sounds were normal. Cardiac and pulmonary examination revealed no pathology. Leukocyte count (9.46x109/L), hemoglobin, hematocrit values and CRP (C reactive protein) and all other biochemical parameters were within normal limits in the laboratory tests performed. Whole abdominal ultrasound revealed an increase in the diffuse wall thickening of the 10 cm-intestinal segment in the right upper quadrant, presumably belonging to colon and an intraabdominal echogenic mass related with this segment in the upper quadrant, midline level. Upon this finding, intravenous contrast-enhanced computed tomography examination was performed. Iliocecal junction was observed in the upper abdomen by CT scan. The colon was the herniated towards the ascending colon pouch. Intussusception at the ileocecal junction and a heterogeneous mass with fat density measuring 40x53x55 mm were observed in this region. There were dense septal appearances in the mass (Figure 1). According to CT scan, lipoma and well-differentiated liposarcoma were considered among the differential diagnosis. Upon these findings, the patient was operated. There was no free fluid in the abdominal cavity during surgery. A luminal mass lesion measuring 4x6 cm was found in the right colon, approximately 5 cm proximal to caecum. It was observed that the mass caused proximal invagination in the proximal colon and the lumen was severely narrowed. No other pathology was detected by inspection and digital examination in the other intraabdominal organs within the field of view. With these findings, right hemicolectomy and end-to-side ileotransversostomy was performed (Figure 2). In the pathological examination of the removed colon segment, the lesion was diagnosed as submucousallipoma in the colon.

## Discussion

Colonic lipomas are uncommon, slow growing, non-epithelial benign adipose tumors of the gastrointestinal tract [1, 7, 8]. Colonic lipoma was first described by Bauer in 1757 [5, 8]. Lipomas are the third most common benign tumor of the intestine following hyperplastic and adenomatous polyps. The incidence of colonic lipomas vary between 0.2 and 4.4% [1, 2]. It is seen more frequently in women aged between 40 and 70 years [9]. Seventy to ninety percent of these tumors are located in right colon. Other locations in descending order include the transverse colon with flexures, the descending colon, the sigmoid colon and rectum. Most often presenting as a solitary mass, multiple colonic lipomas can be seen in 6-25% of the cases [9, 10]. Lipomas have a low recurrence rate after surgical excision and manifest a good clinical course [8]. Size of lipomas varies between 2 mm and 30 cm in the literature [5]. Approximately 90% of colonic lipomas are submucosal, the remaining originate from the subserosal or intramucosal area [1]. Our case was similar to those found in the literature with regard to gender, localization of the mass in colon-submucosal area and that the mass was solitary. Colonic lipomas are usually small and asymptomatic [8]. Therefore, most of the lipomas are detected incidentally during imaging modalities, colonoscopy, surgery or autopsy [2, 3, 10]. They are usually symptomatic when their size is greater than 2 cm [3, 8, 10]. Only 25% are symptomatic and the most common symptoms include abdominal pain, rectal bleeding and change in bowel habits [2, 3, 8]. The main symptom in our patient was abdominal pain. Intussusception is rare in adults and accounts for only 1-5% of all intestinal obstructions. Most cases of intussusception occur due to a benign or malignant neoplasm that causes invagination in the lumen [2, 3].

Lipomas larger than 4 cm are considered giant lipomas and 75% of them are symptomatic [5]. In our case the lesion measured 40x53x55 mm in size and the lipoma resulted in intussusception. Colonic lipomas can be identified by several radiological procedures. Large lesions may be manifested by lucent mass effect, or as intestinal obstruction in plain X-ray radiographs. CT colonography with barium enema may reveal cavity filling defect with well-defined borders [10]. There was no pathological finding in plain X-ray of the abdomen in standing position in our case. The “squeeze sign” finding, which is manifested by elongation of the spherical shaped filling defect that occurs during peristalsis in the barium graphy, is thought to be pathogromic for colonic lipomas. Ultrasound of the abdomen may reveal a hyperechoic lesion without internal vascularity [10]. In our case, lipoma mass was visualized as echogenic lesion in ultrasound examination. Endoscopic ultrasound examination can be performed to assess the extent of penetration into the muscularis propria [10]. Computed tomography reveals an intraluminal mass with uniformly ovoid-shaped sharp borders and characteristic homogeneous fat density (-40 to -120 Hounsfield units) [6, 7, 10]. In our case, CT scan showed that lipoma had the same density as fat tissue (-42 Hounsfield units) but there were dense septae in the lesion. It is difficult to distinguish lipomas radiologically from well-differentiated liposarcomas. Lipomas may have very few thin septa, while well-differentiated liposarcomas have larger, thicker and nodular septa. It is difficult to distinguish them from each other [11]. Therefore, in CT scan, we considered liposarcoma in differential diagnosis. The signal intensity of fat tissue in magnetic resonance imaging and the suppression of fat signal in fat-weighted sequences are typical characteristics of lipomas, therefore MRI is more sensitive in detecting lipomas [8, 10]. Colonoscopy can distinguish colonic lipomas from cancer or other tumors. Colonoscopy allows direct visualization of the submucosallipoma, which appears as a mass covered with normal mucosa, colonoscopy may also show ulcerated or necrotic mucosa covering the lesion [5, 10]. Intussusception requires immediate operation. Segmental resection is an adequate treatment when colonic lipomas are diagnosed prior to surgery [5]. Intussusception was present in our patient and diagnosis of lipoma/well-differentiated liposarcoma was established with CT before the surgery.

## Conclusion

Colocolic intussusception, one of the causes of acute abdomen, is a clinical picture that is usually seen in childhood. Although it is seen rarely in adults, it is mostly due to malign tumors. However, large, colonic lipomas can also cause colocolic intussusception and lead to acute abdomen. For this reason, it should be kept in mind that patients presenting with ileus may have intussusception due to colonic lipoma.

## Competing interests

The authors declare no competing interests.
